# Patients’ experiences with the advanced practice nurse role in Swiss family practices: a qualitative study

**DOI:** 10.1186/s12912-020-00482-2

**Published:** 2020-09-23

**Authors:** Nicole Schönenberger, Beat Sottas, Christoph Merlo, Stefan Essig, Stefan Gysin

**Affiliations:** 1 Institute of Primary and Community Care Lucerne, Lucerne, Switzerland; 2 Department of Health Sciences and Medicine, University of Lucerne, Lucerne, Switzerland; 3 formative works, Fribourg, Switzerland

**Keywords:** Swiss primary care, General practice, Advanced nursing practice, Advanced practice nurse, Nurse practitioner, Primary care nurse practitioner, Patient perspective, Care coordination, Qualitative research

## Abstract

**Background:**

Considering shortages of general practitioners (GP) and strategies for improving the quality of health care provision, many countries have implemented interprofessional care models with advanced practice nurses (APN). International evidence suggests that APN care results in high patient satisfaction. In Switzerland, the role is still new, and the patient perspective has not yet been researched. Our aim was therefore to explore patients’ experiences with the APN role in Swiss family practices.

**Methods:**

We conducted 22 semi-structured interviews in four different family practices with patients aged 18 to 97 suffering from minor acute to multiple chronic diseases, and who had at least one consultation with an APN. All interviews were audiotaped, transcribed verbatim, and analysed using qualitative content analysis.

**Results:**

The analysis resulted in five themes: Despite the unfamiliarity, all patients were willing to be consulted by an APN because it was recommended by their GP (1); after several encounters, most participants perceived differences between the APN and the GP consultation in terms of the length and style of the consultations as well as the complexity of their tasks (2); the interviewees emphasised coaching, guidance, care coordination, and GP-assisting tasks as APN core competencies and attributed the characteristics *empathetic*, *trustworthy*, and *competent* to the APN role (3); most patients especially valued home visits and the holistic approach of the APNs, but they also noticed that in certain cases GP supervision was required (4); and due to the close collaboration between the APN and the GP, patients felt safe, well cared for and experienced improvements in physical and psychological well-being as well as in daily activities (5).

**Conclusion:**

Our results suggested that patients value the APNs’ competencies, despite their initial lack of role knowledge. Trust in the GP seemed to be the most important factor for patients’ receptiveness toward the APN role. Overall, patients perceived an added value due to the enlargement of the scope of practice offered by APNs. The patient perspective might provide valuable insights for further APN role implementation in Swiss family practices.

## Background

Aging and multimorbidity have shifted patients’ health care needs. Most elderly people desire longer consultations with a focus on counselling and guidance in order to maintain high quality of life despite chronic illness [1, 2]. Hence, tasks such as counselling have become more prominent in the daily activities of general practitioners (GP). Simultaneously, it has been debated whether there are enough GPs—especially in rural areas—to meet the growing demand for these services and whether such services exclusively require the expertise of a GP [3–5]. Therefore, many countries, including the United States, Canada, the United Kingdom, the Netherlands or Sweden, have implemented interprofessional care models with advanced practice nurses (APNs) to meet the changing healthcare needs of patients in times of GP shortages, to improve access to care and to enhance continuity of care, especially in primary care [6]. In these countries, the APN role is often specified and mostly referred to as Nurse Practitioner (NP) or Primary Care Nurse Practitioner (PCNP). In Switzerland, the term APN is most common and is therefore used in this paper.

International literature indicates that depending on the country and reason for implementation, APNs provide care for patients with mild acute or chronic diseases. This is done either to free up GPs’ time for more acute and challenging conditions or to focus on complementary tasks to meet the needs of patients that are not sufficiently met by the health care system with its current role configuration [5–7]. APNs tasks include the initial assessment of needs, routine follow-up care and advising patients on self-care management or life changes [3, 8, 9]. Several studies have shown that APNs, regardless of the field of implementation (hospitals, general practice or hospital at home models) are likely to achieve higher patient satisfaction than GPs, due to longer consultations, the comprehensive provision of health information and advice, as well as their psychosocial (patient-centered) communication style [3, 6, 10–13]. In addition, Jakimowicz [14] found in their review, that patients appreciated the involvement of APNs in their treatment because they displayed a focus on the patient as a person with additional concerns and not just on the disease. Despite positive results, many studies indicated that patient satisfaction also depends on characteristics such as age, marital status, education level, overall health status or familiarity with the APN [15–17].

Switzerland is still in the early phases of introducing APNs to primary care. There have only been a handful of pilot projects and hardly any physician–nurse substitution in family practices so far. One reason might be that educational programs have only recently begun to teach APN students the clinical skills and competencies required to work in independent primary care practice, such as physical examinations, clinical reasoning, and pharmacology [18–20]. Further reasons might include the resistance of GPs, the lack of accepted educational standards, clear regulations in terms of scope-of-practice (e.g. prescribing) and the current tariff system in primary care, which was mainly designed for medical doctors [19, 21–23]. For the time being, APNs can only prescribe medications under physician delegation [24].

Even though studies about APNs in Switzerland are limited, there is the so-called PEPPA Plus framework by Bryant-Lukosius et al. [25] for evaluating the impact of the different APN roles. Based on this framework, Gysin et al. [10] explored APNs’ and GPs’ first experiences with introducing the APN role into Swiss family practices. They found that both APNs and GPs agreed on the added value of the APN and highlighted the importance of raising awareness among GPs and implementing suitable regulations to ensure the long-term sustainability of the APN role. There have been other studies about the APN role in general practice [26, 27], but hardly any examined the patient’s perspective, although the PEPPA Plus framework acknowledges that patients’ opinions are decisive in assessing the quality of healthcare provided by APNs [25]. Therefore, we aimed to explore and understand patients’ experiences with the APN role in Swiss family practices. The specific objectives were 1) to assess patients’ acceptance of and satisfaction with the APN role, 2) to describe the perceived differences between APN and GP consultations, and 3) to explore the advantages and limitations of having an APN in family practices from the patient perspective.

## Methods

This study followed an explorative qualitative design with semi-structured, individual patient interviews using qualitative content analysis.

### Setting

Data was collected from four different family practices in central and north-east Switzerland. These practices were selected because they all engaged an APN as part of a pilot project and were researched in larger evaluations [10, 22]. The projects varied in their implementation strategy. In practices A and D, it was mainly the government’s decision to use an APN in a family practice due to the lack of GPs (top-down), while in practice C the GPs employed an APN and managed the implementation process themselves (bottom-up). The project at practice B was an extension of the project at practice A, and engaged the same APN. Table 1 provides an overview of the setting and characteristics of the pilot projects. All APNs had a master’s degree in nursing and many years of practical experience as registered nurses, mainly in stationary settings like hospitals. By the time of the interviews, the APN in practice A had 1 year of experience respectively two in practice B in her role as an APN. The APN in practice C had one and the APN in practice D 2 years of experience in the role. The APNs in practices A/B and D were completing an additional postgraduate training with a clinical mentorship by the GPs to consolidate their application of physical assessments, pathophysiology, and pharmacology during these projects. The mentorship included 900 h of clinical training during which the APN went through phases of observing, supervised performing and unsupervised performing as well as reflection [19]. All APNs provided direct patient care, counselling and physical examinations during in-office consultations and carried out preventive and follow-up home visits to mainly multimorbid elderlies. However, the autonomy of care delivery and the proportion of in-office consultations or home visits varied in each practice and according to the level of training [28].

**Table 1:** Characteristics of the pilot projects

	Practice A	Practice B	Practice C	Practice D
**Location**	Municipality of a small or suburban agglomeration in central Switzerland^a^	Municipality of a small or suburban agglomeration in central Switzerland^a^	Rural peripheral municipality in central Switzerland^a^	Rural, centrally located municipality^a^
**Practice setting**	Traditional family practice with two GPs	Group practice with five GPs	Single practice	Interprofessional practice with six GPs and five other health professionals
**APN**				
**TEP**	50%, declining to 20% (as of January 1, 2019)	40%	30%	50%
**Education**	MSc in Nursing, DAS Complex Care	MSc in Nursing, DAS Complex Care	MSc in Nursing	MSc in Nursing, DAS ANP-plus
**Project**				
**Launch**	August 2017	January 2019	August 2018	April 2016
**Initiator**	Cantonal health department	Practice team	Cantonal health department & cantonal hospital	Practice team
**Implementation strategy**	Top-down	Extension of project in practice A	Top-down	Bottom-up
**Rationales**	Shortages of GPs	Interprofessional task-sharing	Shortages of GPs	Interprofessional task-sharing

*GP* General practitioner, *APN* Advanced Practice Nurse, Other health professionals = psychologists, physiotherapists, nutritionist, occupational therapist; *TEP* Total employment percentage, *MSc* Master of Science, *DAS* Diploma of Advanced Studies, *ANP* Advanced Nursing Practice

^a^ Municipality typology 2012: https://www.atlas.bfs.admin.ch/maps/13/de/12360_12482_3191_227/20593.html

### Sampling strategy and participants

Purposive sampling was used to cover the knowledge, expectations, and opinions of patients on the APN role [29]. Within the ongoing projects, potential patients were selected by the research team in conjunction with the APNs and the GPs. Patients were eligible if they were at least 18 years old, did not suffer from cognitive deficits (e.g. dementia), and had had at least one consultation with the APN. With their prior consent, the interviewees’ contact information was forwarded to the research team by the APNs, whereupon the patients were contacted directly. After 22 patient interviews, data saturation was considered to be achieved as no new ideas or categories appeared in the analysis. Participants were aged 18–97 years (mean = 71; SD = 21.5) and suffered from minor acute to multiple chronic diseases. Thirteen of the participants were female, and nine were male.

### Data collection

All 22 interviews were conducted by members of the research team (NS, SG, BS) between July 2018 and October 2019. Six participants were interviewed in practice A, five in practice B, two in practice C, and nine in practice D. Particular attention was paid to ensuring a comfortable and safe atmosphere, because the researchers and patients did not know each other beforehand. In agreement with the participants, seventeen interviews were conducted either at the family practice or at the interviewees’ homes, and five interviews were conducted by telephone. In two interviews, the spouse was also present at the interview. A semi-structured interview guide was developed to cover aspects considered relevant by the literature and experts, i.e. differences between APN and GP consultations, expectations, tasks, and competencies [3, 8] and to guarantee a consistent structure for all interviews, without restricting the conversation. The guide was adapted to the respective settings (see Additional file 1: Interview guide). All interviews were conducted in Swiss German and lasted between 12 and 62 min. They were audiotaped and transcribed verbatim. To ensure anonymity and a consistent translation from spoken to written language, transcription rules and confidentiality agreements were predefined [29].

### Data analysis and rigor

The data was analysed and interpreted using qualitative content analysis based on Graneheim et al. [30]. This iterative methodology was chosen because it focuses on qualitative nursing research and ensures both trustworthiness and scientific rigor. First, the interview transcripts were read several times in order to obtain a general understanding of the content. The meaning units, which captured the patients’ experiences with the APNs, were highlighted and condensed into brief phrases or single words. These were then used as labels for the codes. In the next step, sub-categories were built with codes that shared commonality, which were then further abstracted into categories. Lastly, different themes were extracted, covering the underlying content of the text (see Additional file 2: Coding structure). All the steps were conducted by the first author (NS) and reviewed by the last author (SG). Disagreements were discussed until consensus was reached. The computer software MAXQDA 2018 (VERBI Software GmbH, Berlin, Germany) served as a supporting tool for the transcription and analysis. We followed the Standards for Reporting Qualitative Research (SRQR) to synthesize and report our study results [31].

## Results

The content analysis resulted in five main themes: 1) openness despite unfamiliarity with the APN role, 2) differences between GP and APN consultations, 3) competencies and characteristics of the APN, 4) added value and limits of the APN, and 5) safety and quality aspects of APN care. Each theme is described in detail below and exemplified by statements from the patients.

### Openness despite unfamiliarity with the APN role

Most patients mentioned that they had no ideas or concepts associated with the term *advanced practice nurse* or knowledge of the training of an APN. They therefore initially saw the APN as an assistant to the GPs, who could take over certain tasks to relieve them.

*“The APN has special training, so she can take over a lot of tasks that otherwise the GP would do.” (Practice B)*

Although patients felt some uncertainty about the APN role, they were generally willing to consult with one because this was recommended and initiated by their GP. The participants emphasized that they had deep trust in their GPs and therefore relied on them to hire only competent health professionals.

*“I simply have complete trust in my GP and thus it seemed right for me.” (Practice B)*

Some interviewees mentioned that their openness to the new role was facilitated by the perceived lack of GPs as well as their personal attitude toward a new and innovative healthcare model.

*“Yes, the lack of GPs is well known, and I think especially here in our canton; it’s important to experiment and look for other solutions because GPs are not easy to replace.” (Practice A)*

### Differences between GP and APN consultation

After getting more familiar with the APN role, the patients noted that the work practices and treatments of GPs and APNs were quite similar.

*“The APN actually did the same examinations as my GP had done yesterday. And asked the same questions in different words, but with the same content.” (Practice A)*

Nevertheless, depending on the nature and complexity of their health problems, patients preferred either the GP or the APN. They considered the medical expertise of a GP to be more comprehensive than that of the APNs. Consequently, all patients attributed more complex and urgent tasks to the area of competence of GPs.

*“It’s another level, so [...] if I seriously had something, [...] I would really say that my GP should come by.” (Practice B)*

In contrast, APNs were more valued for tasks that are known to be time-consuming. Most of the participants stressed that consultations by the APN often lasted 30 to 60 min, whereas GP consultations were usually less than 20 min.

*“The APN just takes the time. She knows that conversations are important. […] Sometimes it only takes half an hour, sometimes an hour, depending on the situation.” (Practice D)*

Patients noticed that all APNs were still supported by the GP, but to varying degrees. One patient compared the situation with medical interns in family practices who needed more support and confirmation at the outset before they develop the skills to carry out tasks on their own.

*“It's actually the exact same situation as with medical interns. […] The young doctors who come from training are not able to decide on their own; the doctor has to supervise them.” (Practice A)*

### Competencies and characteristics of the APN

All patients attributed characteristics like *empathetic*, *pleasant, competent* and *trustworthy* to the APN role.

*“The APN is a competent, calm, and representative woman who can listen to patients. And that’s something […] that immediately made me trust this woman.” (Practice B)*

Patients emphasized that the APN really cared about their feelings and the impact of the disease on their daily lives. They felt that the APN empowered them in their self-management process by giving advice and applying individualized interventions and strategies.

*“The APN gives me tasks and encourages me to solve those tasks somehow. And then I really try to get this under control.” (Practice D)*

Another competency that patients attributed to the APN was the initial assessment of needs. They stressed that the knowledge of the APNs enabled them to assess the urgency of the health problem and thus determine whether an appointment with the GP or even hospitalization were indicated.

*“If the APN has only been there once or twice, then she knows the patients. And when they are ill, [...] she can decide whether the doctor should come immediately or whether an appointment can be made.” (Practice B)*

Many patients reported situations in which the APN referred them to other professional groups, like psychiatrists, or to the notary’s office to draw up a will. Another patient mentioned that, after mutual agreement, the APN had put her in touch with a peer.

*“I go to a psychiatrist; my APN recommended him to me because I told her that […] these relaxants were no longer of any use.” (Practice D)*

From the patients’ perspective, the APN also took over “medical” tasks such as taking a medical history, reviewing lab results and performing physical examinations as these tasks are usually performed by GPs and not by medical assistants or registered nurses in Swiss family practices.

*“When the APN comes by […], she asks how it’s going and then she says what she needs to do. Let’s say that she has to take blood or that she has to listen to the heart and lungs or those things ...” (Practice B)*

Additionally, participants emphasized that the APNs advised them on questions regarding medication intake and made minor changes. A few appreciated that the APNs offered alternatives, such as natural remedies or nutritional advice to minimize medicalization.

*“[...] the APN suggested sage drops and explained to me what they were for and when I should take them.” (Practice D)*

### Added value and limits of the APN

Patients described the APN as a reference person with whom they felt a close relationship and whom they dared to ask questions they would not have asked the GP. They particularly experienced the added value from the holistic care provided by the APN.

*“I experience her as a person who has a broad knowledge and who looks at you as a person, how you really are, and also at the environment.” (Practice D)*

Nevertheless, patients appreciated being treated by the APN and GP at the same time. In their view, both had different perspectives but still pursued the same treatment goals.

*“With the APN, I rather have other problems to solve (pause) I suppose […] often there are private things involved that have an effect. And there you get more personal and closer to the APN than you would ever dare with a GP.” (Practice D)*

Multimorbid and elderly patients saw home visits as an enormous relief in terms of organizational and physical effort. In addition, they felt more comfortable at home, making it easier for them to open up and engage with the APN.

*“If I have my sessions in the practice, the APN does not experience me as I am at home. You open up differently at home, you also play a different role.” (Practice D)*

Regarding the health system, a few patients drew the conclusion that the APN consultations would probably be cheaper than GP consultations and thus lead to lower healthcare costs. Others mentioned concerns about the workload and shortage of GPs and suggested that the APN could be an additional resource to ensure continuity of care.

*“I think it is a great experiment and probably also a necessary step to relieve GPs […] by trying to go multi-track with advanced practice nurses.” (Practice A)*

On the other hand, some patients from Practices A, B, and C were reluctant to allow the APN to play the leading role in their care and preferred to receive additional confirmation from their GP for the diagnosis and/or treatment of acute minor or chronic diseases.

*“The important thing is that you can still talk to the GP when the APN does the examination.” (Practice A)*

As a result of such double-checks by the GPs, a few patients reported waiting times during the consultation, which raised questions about the efficiency of the role and the organization within the practice.

*“I don't think it’s quite organized yet […]. I think also, for GPs […], they have patients themselves and then there are ‘in-between patients’ […]. I don’t know what it’s like for GPs, if it gets even more stressful for them…” (Practice A)*

### Safety and quality aspects of APN care

Patients’ safety assurance was often mentioned when talking about the new role and the scope of practice of the APNs. However, the interviewees felt well cared for, partly because of the close collaboration between the GP and the APN, and partly because they realized that the APNs could provide greater continuity of care and were able to assess their own limits and not exceed them. No participant mentioned complications from treatment or moments of uncertainty.

*“I think, if I said, ‘Please call the doctor,’ then the APN would do it immediately. I don't get the feeling that she wants to play doctor in the first place.” (Practice B)*

All patients emphasized that they were satisfied with the APN consultations. Most reported improvements in psychological well-being, daily activities, or symptoms, such as pain or insomnia, due to the interprofessional care provided by APN and GP.

*“With the APN, it’s like when you get a pill and then all of a sudden you feel good.” (Practice D)*

*“I had a time when I didn’t feel so well because of the pain and sleeping problems. There she was, just insanely good to me, she just helped me.” (Practice D)*

Patients felt that the new role added value to the practice, but also emphasized that the APN and GP were equally important and that the aim was not to replace one profession but for them to complement each other.

*“And then I said to the GP, ‘This APN is so good; you could still recruit a dozen more.’”*

*(Practice B)*

## Discussion

### Summary of the results

The analysis of the 22 semi-structured interviews resulted in five themes. Despite the unfamiliarity, patients were willing to be consulted by an APN because it was recommended by their GP (1: openness despite unfamiliarity with the APN role). After several encounters, the participants perceived differences between the APN and the GP in terms of the length and style of the consultations as well as the complexity of their tasks (2: differences between GP and APN consultations). The interviewees stressed coaching, guidance, care coordination, and GP-assisting tasks as APN core competencies and attributed the characteristics *empathetic*, *trustworthy*, and *competent* to the role (3: competencies and characteristics of the APN). The patients especially valued home visits and the holistic approach of the APNs, but they also noticed that in certain cases GP supervision was required (4: added value and limits of the APN). Due to the close collaboration between GP and APN, patients felt safe, well cared for and experienced improvements in physical and psychological well-being as well as in daily activities (5: safety and quality aspects of APN care).

### Interpretation of the results

In all four practices, the initial unfamiliarity of the patients with the APN role confirmed that Swiss primary care is still in the early phases of role implementation. However, the lack of knowledge did not appear to be a decisive factor for patients regarding whether or not to be consulted by an APN. Instead, patients were generally open-minded and trusted their GPs when they suggested an APN consultation. Interestingly, it was not the education nor the title that was important for patients in accepting the role, but rather their competencies, skills and behaviour. In addition, comprehensible rationales for introducing APNs (e.g. GP shortages, length of consultations, home visits) have facilitated patients’ openness and acceptance of the new role.

Depending on the practice and implementation strategy chosen for the projects (top-down or bottom-up), different priorities emerged. In practice D, the APNs’ focus of care was mainly on the holistic treatment of chronically ill patients, whereas in the other practices, APNs covered a broader scope of patients’ healthcare needs, including the diagnosis and treatment of minor acute and chronic diseases. In practices A, B and C, patients noted that the APNs required more supervision but were able to reduce the GPs’ workload to a greater extent. However, it is possible that the APNs’ autonomy of care delivery was not only influenced by the task allocation within the practice, but also by the varying levels of training and practical experience of the different APNs.

**Fig. 1:**
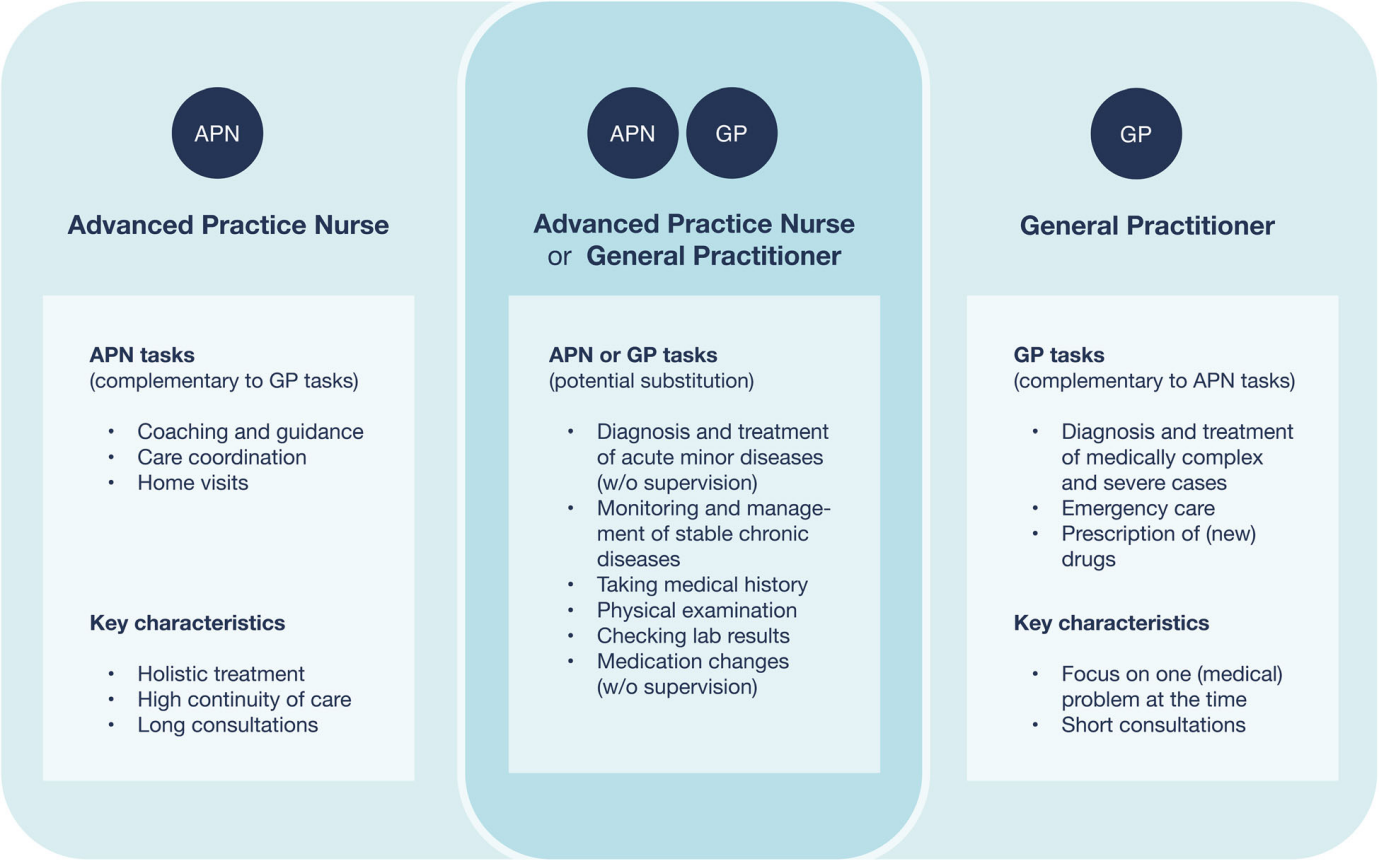
Differences between the APN and GP perceived by patients after becoming familiar with the APN-role

Patients highly regarded the enlargement of the scope of practice offered by APNs. They were able to build closer relationships with their patients than GPs, most likely due to longer consultations, a holistic approach, and their communication styles. However, when the symptoms and complaints were classified as medically severe, patients were reluctant to allow the APN to play the leading role in their care and preferred to consult their GP instead. Patients appreciated having both the APN and GP involved in their treatment, as they saw the APN perspective as complementary to that of the GP, and thus experienced an increase in care quality through the collaboration. The differences between the APN and GP perceived by patients are illustrated in Fig. 1.

### Comparison with other studies

A review by Laurent et al. [3] revealed that most patients do not know the exact role or background of their care provider and instead judge them based on their competencies. These findings were largely consistent with ours, but the APN role has been recently implemented in Swiss family practices, and the patients initially did not have knowledge about their competencies, which resulted in some uncertainties. Redsell et al. [32] confirmed that initial uncertainty is a common feature of the implementation phase and often influences patients’ preferences for being consulted either by a GP or an APN. In contrast to our findings, Williams et al. [33] explained that patients’ preferences for their healthcare provider were based not only on the perceived severity of the health problem, but also on time—time to discuss problems or the time saved by solving problems. In a study from 2019, Gysin et al. [10] stated that APNs in Swiss family practices best meet the needs of elderly, multimorbid and complex patients. Contrary, in our study the patients preferred to consult their GPs in complex cases. Cody et al. [34] noted in their study that the concept of complexity in primary care remains inconsistent, but there is a slight tendency for APNs to treat socially complex patients, while GPs focus on medically complex cases. The patient statements in our study could therefore refer to medical complexity.

In countries where the APN role is well established, evidence suggests that APNs achieve at least equally good health outcomes for patients with chronic diseases as GPs do [8]. Van der Biezen et al. [5, 35] indicated that APNs also make a valuable contribution to the treatment of patients with acute problems. The patients in their studies stated that APNs could provide both chronic care and acute care of minor illnesses, but the latter under GP supervision. In our study, however, APNs often sought the advice of a GP for patients with chronic and mild acute illnesses. Patients were therefore unsure whether the APN role leads to efficient results and whether the introduction of APNs in family practices could stabilise or reduce cost, as mentioned in the review of international studies by Delamaire et al. [6]. The hospital at home model, in which APNs were involved, also reported positive results in terms of efficacy, costs and patient satisfaction [11]. However, Delamaire et al. [6] stated that if APNs take on supplementary tasks, it often results in higher cost According to van der Biezen et al. [5], the decision whether to focus on substitutive or complementary tasks depends largely on the GPs’ motives for hiring an APN. In our study, we found that in practices where the APNs were employed due to a lack of GPs patients identified task substitution more frequently, while in practices that were looking for an innovative interprofessional care model complementary tasks were reported more often. Regardless of the practice, the patients confirmed having longer consultations and more holistic treatment with APNs than with GPs. These have also been mentioned in other studies as primary features of APN care [10, 14, 36].

### Limitations

The number of interviews conducted in each practice varied as it proved difficult to find willing participants who met the inclusion criteria in some practices. Additionally, the APNs and GPs were involved in the selection of the patients, and therefore, we cannot exclude selection bias. However, we conducted interviews in four different practices, and we achieved a comprehensive sample of patients from different places of residence, with a wide range of ages, and with different health conditions. The generalizability and transferability of our results might be limited because the levels of education and experience of the APNs varied, as did the length and development of the projects. Furthermore, we did not have quantitative information regarding consultation times, the assessments done by the APNs and their autonomy in care delivery. Lastly, our study may be prone to researcher bias, because only one person coded the transcripts. However, the analysis followed the strict steps of the chosen methodology and each step was extensively discussed by two reviewers to ensure trustworthiness.

### Implications and outlook

Promoting the APN role among GPs could facilitate the implementation of APNs, since the openness of patients largely depended on the trust and recommendation of their GPs [14, 37]. Additionally, a future APN role description should provide comprehensible rationales for the implementation of the role and should not simply imply that an APN is a GP’s assistant. For further role development in Switzerland, more small- and large-scale pilot projects are important to determine the scope of practice and the target patient population of APNs. Irrespective of whether APNs focus on complementary or substituting tasks, it is important to distinguish the APN’s area of responsibility and competencies from that of a GP. Patients should be able to recognize the differences between APNs and GPs, and experience benefits from the introduction of the APN role. Furthermore, clear guidelines must be developed to enhance the efficiency of the process as well as patient safety. Our study provides valuable first insights into the patient perspective for further pilot projects. It will be important to further validate and quantify the quality of care and the efficiency of the skill mix when including an APN in a family practice.

## Conclusion

Our results suggested that patients value the APNs’ competencies, despite their initial lack of role knowledge. Trust in the GP seemed to be the most important factor for patients’ receptiveness toward the APN role. Overall, patients perceive an added value due to the enlargement of the scope of practice offered by APNs. The patient perspective might provide valuable insights for further APN role implementation in Swiss family practices.

## Supplementary information

**Additional file 1.** Interview guide.

**Additional file 2.** Coding structure.

## Data Availability

The data generated and analysed during the current study are not publicly available due to the sensitivity of the data (contains patients’ health information). Parts of the transcripts are available from the authors upon reasonable request and with permission of the study participants.

## Abbreviations

GP: General Practitioner
APN: Advanced Practice Nurse
