# Supplementary material for: Patients’ experiences with the advanced practice nurse role in Swiss family practices: a qualitative study
Source: BMC Nurs. 2020 Sep 23;19:90. doi: 10.1186/s12912-020-00482-2 (PMC7510323; doi:10.1186/s12912-020-00482-2)
Supplement: Supplementary file 1 — Additional file 1. Interview guide. [file 12912_2020_482_MOESM1_ESM.docx]

**Additional file 1: Interview guide**

Interview guide for patients

1. In general, why do you visit the family/group practice and what are your expectations and goals for these visits?
2. What did you think when you heard that an *APN* would take care of you, and not the GP himself/herself?
3. What expectations did you have about the (1st) consultation with the *APN*?
4. In the case of home visits: For what reasons does the *APN* make home visits to you?
5. What is your preferred location for an *APN* consultation (in practice, at your home, at the elderly home) and why?
6. Can you describe a typical appointment with the *APN*? What did the *APN* specifically do?
7. Was the GP also involved in your consultation and, if so, for what reasons?
8. How satisfied are you with the consultations and treatment by the *APN*?

Which of your expectations were met? Which ones less?

In your opinion, was the *APN* making the right health decisions for you?

How safe did you feel before/during/after your treatment?

Did you feel comfortable with the *APN*?

1. What recommendations did you receive from the *APN* and what did you do with them? Do you have a concrete example of what has helped you in particular?
2. What do you think the *APN/GP* is particularly good at?
3. What do you think falls more within the scope of practice of *APNs/GPs*?
4. How does a consultation with the *APN* differ from a consultation with a *GP*?
5. How do you rate the professional knowledge of the *APN*?
6. Where do you see the *APNs*’ strengths?
7. Would you consider consulting the *APN* again in the future? In which cases, yes? In which cases, not?
8. Would you recommend the *APN* to your colleagues/family etc.? If so, why?
9. Is there anything else you would like to add?
