# Supplementary material for: Patients’ experiences with the advanced practice nurse role in Swiss family practices: a qualitative study
Source: BMC Nurs. 2020 Sep 23;19:90. doi: 10.1186/s12912-020-00482-2 (PMC7510323; doi:10.1186/s12912-020-00482-2)
Supplement: Supplementary file 2 — Additional file 2. Coding structure. [file 12912_2020_482_MOESM2_ESM.docx]

**Additional file 2: Coding structure**

| **Theme 1** | **Openness despite unfamiliarity with the APN role** | | |
| --- | --- | --- | --- |
| **Category** | Unfamiliarity with the APN role | Facilitating factors | |
| **Subcategory** | Beliefs about the APN role | GP-related factors | Need for redesigning care models |
| **Codes** | - Received information about the new role | - Trusting relationship | - Shortage of GPs - Personal attitude |

| **Theme 2** | **Differences between GP and APN consultation** | | | | |
| --- | --- | --- | --- | --- | --- |
| **Category** | Reason for consultation | Content of consultation | Setting | Time aspects | Level of Supervision |
| **Subcategory** | Severity and complexity of the symptoms | NA | NA | NA | Knowledge and skills |
| **Codes** | - Disease history - Urgency | - Conversation - Clinical examination - Treatment and care | - Home visits - In-office consultation | - Duration - Continuity of care - Waiting times | - Work experience - Education |

| **Theme 3** | **Competencies and characteristics of the APN** | | | |
| --- | --- | --- | --- | --- |
| **Category** | Core competencies | | | Personality characteristics |
| **Subcategory** | Coaching and Guidance | Care coordination | GP assisting tasks | Appearance and behaviour of the APN |
| **Codes** | - Providing information and advices - Patient empowerment - Individual action planning | - Initial assessment of needs - Referral to other health professions | - Physical examination - Diagnostic measures - Control of a safe medication and minor changes - Follow-up consultation | - Trustworthy - Pleasant - Empathic - Competent - Serious - Transparent - Flexible |

| **Theme 4** | **Added value and limits of the APN** | | |
| --- | --- | --- | --- |
| **Category** | Utility | | Limitation |
| **Subcategory** | Added value for patients | Benefits for the health care system | NA |
| **Codes** | - Reference person - Holistic treatment - Time resources - Home visits - Joint treatment goals | - Work relief (GP) - Less health care costs | - Organizational aspects - Duplicity of tasks - Uncertainty |

| **Theme 5** | **Safety and quality aspects of APN care** | |
| --- | --- | --- |
| **Category** | Feeling of security | Patient reported outcomes |
| **Subcategory** | Interprofessional collaboration | Functionality and quality of life |
| **Codes** | - Interprofessional communication - Awareness of own limits and competencies - Personal choice of provider | - Satisfaction with care - (Potential) recommendation to others - Improvement of symptoms - Improvement of activities of daily living¨ - Improvement of psychological well-being |

*GP = General practitioner; APN = Advanced Practice Nurse; NA = not applicable*
